# Study protocol: Traditional Chinese Medicine (TCM) syndrome differentiation for heart failure patients and its implication for long-term therapeutic outcomes of the Qiliqiangxin capsules

**DOI:** 10.1186/s13020-021-00515-1

**Published:** 2021-10-16

**Authors:** Alice Yeuk Lan Leung, Hoiyong Chen, Zhenhua Jia, Xinli Li, Jiangang Shen

**Affiliations:** 1 School of Chinese Medicine, University of Hong Kong, 10 Sassoon Road, Pokfulam, Hong Kong, People’s Republic of China; 2 National Key Laboratory of Collateral Disease Research and Innovative Chinese Medicine, Shijiazhuang, China; 3 Hebei Yiling Hospital, Key Disciplines of State Administration of TCM for Collateral Disease, Shijiazhuang, China; 4 Department of Cardiology, The First Affiliated Hospital with Nanjing Medical University, Guangzhou Road 300, Nanjing, 210029 China

**Keywords:** Qiliqiangxin (QLQX) capsule, Traditional Chinese Medicine (TCM), TCM syndrome differentiation, Syndrome, Syndrome element, Tongue diagnosis, Syndrome questionnaire, Chronic heart failure, Treatment efficacy

## Abstract

**Background:**

Syndrome differentiation is a commonly used methodology and practice in Traditional Chinese Medicine (TCM) guiding the diagnosis and treatment of diseases including heart failure (HF). However, previous clinical trials seldom consider the impact of syndrome patterns on the outcome evaluation of TCM formulae. Qiliqiangxin (QLQX) capsule is a TCM formula with cardiotonic effect to improve the cardiovascular function for heart failure with proven efficacy from well-designed clinical trials. Though, there is no clinical trial with a large sample size and long assessment period that considers the relationship between TCM syndrome differentiation and the treatment efficacy of QLQX. In the present study, we design a study protocol to evaluate the relationship between TCM syndrome differentiation and the severity of heart failure as well as its progression. Furthermore, we will evaluate the impact of the TCM syndrome patterns on the efficacy of QLQX in the outcome of heart failure.

**Methods:**

This is a clinical study conducted in conjunction with an ongoing clinical trial (QUEST Study) by sharing the parent patient populations but with different aims and independent designed roadmaps to investigate the TCM syndrome pattern distributions and the impacts of syndrome pattern types on the efficacy of QLQX in HF treatment. The clinical trial involves over 100 hospitals in mainland China and Hong Kong SAR with 3080 HF patients. By assessing the morbidity and re-hospitalization, we will verify and apply a modified TCM Questionnaire to collect the clinical manifestations of HF and acquire the tongue images of the patients to facilitate the syndrome differentiation. We will base on the “2014 Consensus from TCM experts on diagnosis and treatment of chronic heart failure” to evaluate the TCM syndromes for the patients. A pilot study with at least 600 patients will be conducted to evaluate the reliability, feasibility and validity of the modified TCM questionnaire for syndrome differentiation of HF and the sample size is calculated based on the confidence level of 95%, population size of 3080 and 5% margin of error. Secondly, we will investigate the characteristic of TCM syndrome distribution of HF patients and its correlation with the functional and biochemical data. Furthermore, we will evaluate the relationship between the TCM syndrome patterns and the efficacy of QLQX in the treatment of heart failure. Lastly, we will investigate the implication of tongue diagnosis in the severity and therapeutic outcome of HF.

**Expect outcomes:**

To our knowledge, this is the first large scale clinical trial to evaluate the impacts of TCM syndrome differentiation on the progression and therapeutic outcome of HF patients and explore the diagnostic value of TCM Tongue Diagnosis in HF patients. We expect to obtain direct clinical evidence to verify the importance of TCM syndrome differentiation for the diagnosis and treatment of HF.

*Trial Registration:* The trial was registered at Chinese Clinical Trial Registry, http://www.chictr.org.cn. (Registration No.: ChiCTR1900021929); Date: 2019-03-16.

## Background

Heart Failure (HF) is an end-stage cardiovascular condition characterized by structural or functional cardiac impairments of ventricular filling or blood ejection, leading to abnormal hemodynamics, activating neuro-hormones, and developing myocardial remodeling [1, 2]. The typical clinical manifestations include shortage of breath, fatigue and ankle swelling, accompanied by peripheral edema and elevated jugular venous pressure, etc. [3, 4]. HF is one of the leading causes of death globally [5]. There are approximately 64.3 million patients with heart failure worldwide [6]. In the US, there are around 5.7 million HF patients and it is estimated to reach 8 million HF patients in 2030, accounting for a 46% increase in prevalence [7]. Around 30–40% of HF patients have a history of hospitalization due to HF, and the reported 5-year all-cause readmission, readmission for HF, and mortality rates are 80.4%, 42.3%, ad 75.4% respectively [8, 9]. Thus, HF becomes a major public health burden in human diseases.

Current therapeutic strategies for HF include the following catalogs: Using nitrates and hydralazine to resolve vasoconstriction; Using angiotensin-converting enzyme (ACE) inhibitors, angiotensin II receptor blockers (ARBs), beta-blockers, and mineralocorticoid antagonists (MRAs) to target neuro-hormonal systems; Using Invabradine to target the sinus nodes discharge and slow down the heart rates for the HF patients with unsatisfactory results with beta-blocker; Using cardiac resynchronization therapy (CRT) targeted the sinus rhythm and implantable cardioverter-defibrillator (ICD) targeted patients with cardiac arrhythmia, etc. [5]. Even with the advanced treatment approaches developed, the outcome of HF treatments is still unsatisfactory as the re-hospitalization of worsening heart failure and mortality remain high.

Traditional Chinese Medicine (TCM) formulae, including Qiliqiangxin, Nuanxin, Shencaotongmai, Yangxinkang, appears to be the effective adjunct therapies with conventional drugs to improve cardiac function of HF patients [10]. According to the TCM theory of Collateral Disease, the heart-qi and/or heart-yang deficiency could induce blood stagnation and the disruption of body fluid metabolism [11]. The HF-induced hemodynamic dysfunctions and edema could be attributed to the pathological status of heart-qi deficiency accompanied with blood stagnation and body fluid accumulation [12]. Syndrome differentiation is a unique TCM diagnosis in guiding therapeutic strategies for heart failure. However, there is no standardized methodology for syndrome differentiation of heart failure [13–15]. The diversities of syndromes lead to difficulties in drawing conclusive outcomes and therefore, hindering the development of TCM treatment for heart failure [16, 17]. On the other hand, Tongue Diagnosis is crucial for identifying syndromes and evaluating the therapeutic responses in TCM practice. Tongue Diagnosis has been applied for different diseases such as metabolic syndrome, cancer, menstrual clinical symptoms, etc. [18–21]. Recent studies observed the correlation between tongue patterns and risk factors of heart failure in dialysis patients [22–24]. The tongue changes were also observed in heart failure patients with normal ejection fraction [22–24]. Whether heart failure patients have specific tongue patterns remains unclear. Therefore, we design this study to explore the impacts of TCM syndrome patterns and tongue features on cardiovascular functions and the therapeutic outcome of heart failure.

Qiliqiangxin (QLQX) capsule is a patent TCM formula approved by China Food and Drug Administration for chronic heart failure [25]. QLQX capsule is extracted from 11 TCM herbs including Astragali Radix, ginseng radix at rhizoma, aconite lateralis radix preparata, salvia miltiorrhizae radix et rhizome, semen descurainiae lepidii, alismatis rhizome, polygonati odorati rhizome, cinnamomi ramulus, carthami flos, periploca cortex, and citri reticulatae periarpium. Previous studies indicate that: (1) QLQX could attenuate cardiac remodeling via activating peroxisome proliferator-activated receptor-γ (PPAR-γ) and mTOR; (2) QLQX could prevent cardiomyocytes hypertrophy via activating PGC-1α and downregulating MiR-199a-5p, etc. [26–30]; (3) QLQX could improve ejection fraction and attenuates the left ventricular remodeling via inhibitory effect of the renin–angiotensin–aldosterone system (RAAS) [31, 32]; (4) QLQX could inhibit the myocardial inflammation and cardiomyocyte apoptosis to promote the proliferation of cardiomyocyte [33]. A multi-center, randomized, double-blind and placebo-controlled clinical trial reported that QLQX, as an adjunct therapy, reduced the level of NT-proB-type natriuretic peptide (NT-proBNP) and improved the NYHA functions, left ventricular ejection fraction (LVEF), 6-min walking distance, quality of life and composite cardiac events in HF patients [34]. Those studies suggest that QLQX is a promising TCM formula for HF patients. However, the efficacy of QLQX on hard endpoint incidents such as re-hospitalization of worsening HF and mortality is yet to be proven. Particularly, whether different TCM syndrome patterns impact the therapeutic outcomes of QLQX is unknown. As such, we have designed an QUEST study [35] to explore the long-term efficacy of QLQX in addition to conventional heart failure treatment of CHF. The primary outcome will be the occurrence of the composite endpoint which is defined as either cardiovascular (CV) death or re-hospitalization due to the exacerbation of HF. The secondary outcome measures include the all-cause mortality, secondary endpoint events (treatment terminated due to worsening heart failure, successful resuscitation after cardiac arrest, malignant arrhythmia, non-fatal stroke), CV death and re-hospitalization due to worsening heart failure in patients with ischemic heart disease, and the level of Serum NT-proBNP. However, this clinical trial only focuses on the efficacies of QLQX on the primary and second outcome without testing the impacts of TCM syndrome patterns on the therapeutic outcome. Furthermore, there is limited investigation to the relationship between tongue diagnosis and therapeutic outcome for HF treatment in literature. As the large-scale clinical trial (QUEST) involves 3,080 patients from over 130 hospitals, this creates a golden opportunity for us to leverage this study and develop a connected but independent clinical trial with completely different research aims and roadmaps. Thus, in the present study, through benchmarking the QUEST, we aim to explore the roles of TCM syndrome differentiation in the disease progression of HF and the therapeutic outcome of QLQX. The study will be coached by three specific objectives to answer the following questions: (1) Whether TCM syndromes are associated with the severity of heart failure; (2) Whether different TCM syndrome patterns have different therapeutic outcomes in QLQX treatment for heart failure; and (3) Whether TCM tongue diagnostic characteristics could be an effective index for the severity of heart failure and the indication of the progression as well as the therapeutic outcomes.

### Methods/design

### Patient recruitment and selection criteria

This study will be carried out with approximately 131 centers located in China and Hong Kong SAR. These centers are eligible hospitals or affiliated hospitals of the medical universities. The target number of patient recruitments will be 3,080, which is calculated according to the PARADIGM-HF study based on the cardiovascular death or hospitalization rate for heart failure [36]. This is an event-driven study and all the recruited patients will remain in the study (whether taking the study drug or not) until the number of the primary endpoint events reaches the predicted target of 620 cases, or the study meets the pre-defined efficacy or safety criteria assessed by the Clinical Event Adjudication Committee (CEAC).

Recruitment of patients will be assessed according to the inclusive and exclusive criteria listed in Table 1. These patients must be at least 18 years old fulfilling all the inclusion criteria including the NT-proBNP, LVEF, NYHA cardiac functional grading, and the standardized drug treatment for at least 2 weeks prior to enrollment, etc.

**Table 1:** Inclusive and exclusive criteria

*Inclusion criteria*
1) Provision of signed informed consent prior to any study specific procedures
2) Male or female, aged ≥ 18 years at the time of consent
3) Established documented diagnosis of heart failure for at least three months ago according to “Chinese Heart Failure Diagnosis and Treatment Guideline” issued by the Chinese Medical Association Cardiovascular Branch
4) Left ventricular ejection fraction (LVEF) ≤ 40% (echocardiogram, radionuclide, ventriculogram, contrast angiography or cardiac MRI)
5) NYHA cardiac functional grading II to III, with stable clinical symptoms; including those diagnosed as grade IV within 2 weeks before enrollment
6) Serum NT-proBNP ≥ 450 pg/ml
7) Those who have received standardized baseline treatment regimens without doses adjusted and given intravenously for at least two weeks prior to enrollment; standardized drug treatment includes: angiotensin-converting enzyme inhibitor (ACEI) or angiotensin receptor blocker (ARB) or angiotensin receptor neprilysin inhibitor (ARNI), beta blocker, and aldosterone receptor antagonist (the optimal therapeutic dose should be achieved, except for contraindications or intolerance)
*Exclusion criteria*
Patients should not enter the study if any of the following exclusion criteria are fulfilled
1) Heart failure caused by valvular disease, congenital heart disease, pericardial disease, arrhythmia or non-cardiogenic disease, or caused by vital organ failure (such as renal, hepatic failure, etc.); and right heart failure caused by pulmonary or other definite causes; and acute heart failure
2) Coronary revascularization (percutaneous coronary intervention [PCI] or coronary artery bypass grafting [CABG]) or cardiac synchronization therapy planned to undergo after randomization, or had received cardiac resynchronization therapy prior to enrolment
3) Any condition outside the CV diseases such as but not limited to malignant tumor, severe mental illness, hematopoietic diseases, neuroendocrine system disease, liver transaminase and alkaline phosphatase ≥ 3 × upper limit of normal (ULN), abnormal renal function serum creatinine > 2 mg/dl (176.82 umol/L), potassium > 5.5 mmol/L
4) Patient with left ventricular outflow tract obstruction, myocarditis, aortic aneurysm, aortic dissection, or obvious hemodynamic changes caused by unrepaired valve
5) Cardiogenic shock, uncontrollable malignant arrhythmia, sinus or atrioventricular block at second degree type II or above without pacemaker treatment, progressive unstable angina pectoris or acute myocardial infarction
6) Uncontrolled hypertension systolic blood pressure (SBP) ≥ 180 mmHg and/or diastolic blood pressure (DBP) ≥ 110 mmHg; or SBP < 90 mmHg and/or DBP < 60 mmHg
7) Participation in another clinical study with an IP during the last month prior to enrolment
8) Women of child-bearing potential (i.e., those who are not chemically or surgically sterilized or who are not post-menopausal) who are not willing to use a medically accepted method of contraception that is considered reliable in the judgment of the investigator, from the time of signing the informed consent throughout the study and 4 weeks thereafter, OR women who have a positive pregnancy test at enrolment or randomization OR women who are breast-feeding
9) Allergic constitution; known to be allergic to research drug
10) Inability of the patient, in the opinion of the investigator, to understand and/or comply with study medications, procedures or any conditions may render the patient unable to complete the study

Before the recruitment, patients must be “clinically stable” by receiving at least two weeks of standardized medication treatment according to the local treatment guideline for heart failure with fixed drug type and dosage, as well as have not taken any intravenous anti-HF drug nor oral TCM medication with similar composition as the QLQX capsules. Once the patients are clinically stable, they will be assigned to the QLQX group or the control group in a ratio of 1-to-1 through the double-blind computerized randomize allocation. The patient will be given either QLQX capsules or placebo capsules during the assessment period in addition to their standard treatment for heart failure following the guidelines for diagnosis and treatment of HF in China 2018 [25] or local guideline. The QLQX capsules and placebo are identical in size and shape. The dosage used in this study is 4 capsules of QLQX or placebo 3 times daily. During the assessment period, patients should not take any TCM or herbs which have similar contents to the QLQX capsule.

**Fig. 1:**
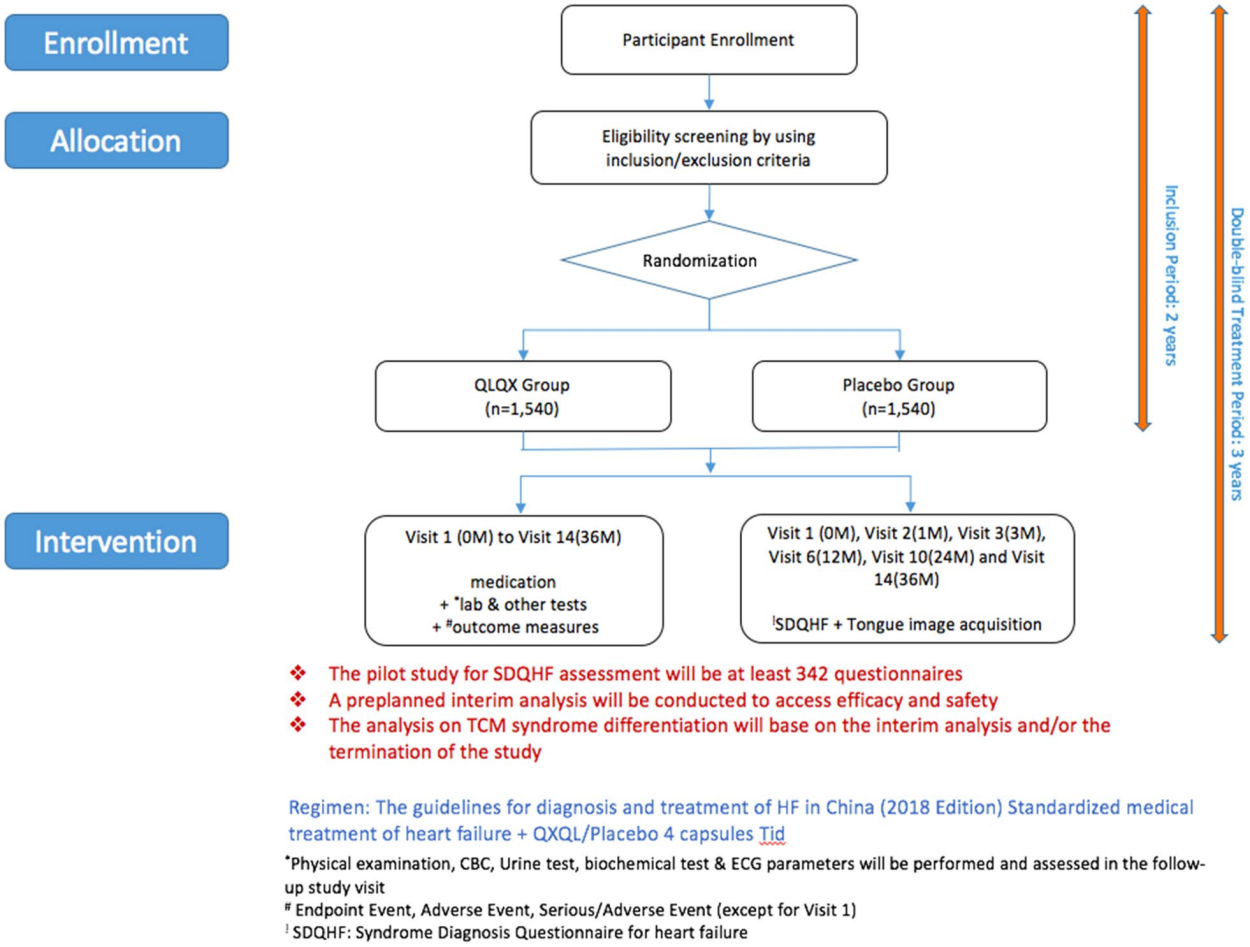
Study flow chart

The entire study will last for approximately 36 months with the first 24 months as the recruitment period. The study will be terminated once the last patient has finished the 12-months assessment. Once recruited, the patient is required to visit the hospitals for efficacy and safety assessment according to the study schedule. All patients are required to sign the Informed Consent Form before the recruitment. For each visit, the patient will be assessed by the researchers/physician. A modified TCM Questionnaire will be used to collect the clinical symptoms and body signs of the HF patients and the tongue images will be acquired from the patients to facilitate the syndrome differentiation. A range of clinical information and parameters will be collected and tests will be performed for the assessment of TCM syndrome, the treatment efficacy, and safety including laboratory and ECG results, etc. The assessment of safety of this study will base on the reporting of the adverse events, SAEs, and laboratory abnormalities, which are closely monitored by the Data Safety Monitoring Committee (DSMC). Two interim efficacy analyses will be conducted when 1/2 and 2/3 primary endpoint events (i.e. around 310 and 414 patients respectively) are gathered. Patients are allowed to withdraw from the study for any reason but the reason is required to be recorded in the case report form. If the patient has an intolerable adverse event that is related to the study drug based on the researcher’s judgment, the patient should immediately discontinue the treatment with the study drug. The study flowchart is shown in Fig. 1.

### TCM Syndrome Diagnosis Questionnaire for Heart Failure (SDQHF)

To collect clinical information for TCM syndrome differentiation, a specialized TCM Syndrome Diagnosis Questionnaire for Heart Failure (SDQHF) is developed according to the TCM syndrome scales for heart failure reported by previous studies [37–40]. The clinical manifestation including the signs and symptoms of heart failure diagnosis which are described according to the “2012 Guiding Principles for Clinical Study on New Drug for Use in Traditional Chinese Medicine”, “2014 Consensus from TCM experts on diagnosis and treatment of chronic heart failure”, “2017 Guideline for TCM Diagnosis and Treatment of Heart Failure (Chronic Heart Failure)” [16, 41, 42].

In the SDQHF, there are 36 closed-ended items and each item is given a ranked scale with simple descriptions to indicate the features of the severity or frequency of signs or symptoms. These 36 items are carefully considered to incorporate the major and concomitant signs/symptoms which are essential for the diagnosis of HF and the TCM syndrome differentiation. The descriptive scale per item is clearly defined and the scales were input by a group of experienced clinical experts and physicians. The SDQHF has gone through the review and pre-pilot testing by 20 subject matter experts including physicians and TCM practitioners.

The 36-items are divided into several dimensions including major signs and symptoms of heart failure, cold and heat characteristics, perspiration, head and general body condition, diet, etc. to facilitate the differentiation of heart failure and TCM syndrome. The terminologies used are specially considered to avoid jargon so that the questionnaire can be easily understood by the researchers/physicians and the patients. It will be an interviewer-administered questionnaire so that unclear questions can be clarified to the patients and ensure patients answer all the questions. The first 31 items in the SDQHF are required to be answered by the patients, and the last 5 items about the physical signs of the patients should be determined by the physician/researcher based on their observation of the patients. The list of 36-items in SDQHF is shown in Table 2.

**Table 2:** The list of 36-items in the TCM Symptom Diagnosis Questionnaire for Heart Failure (SDQHF)

1. Palpitations	10. Coughing out of phlegm	19. Cold sweat	28. Constipation
2. Chest tightness	11. Color of phlegm	20. Fear of cold	29. Diarrhea
3. Precordial pain	12. Texture of phlegm	21. Coldness feeling in upper or lower limbs	30. Frequency of urination
4. Dizziness	13. Feeling fullness in chest/abdomen	22. Feeling heat in 5 centers	31. Amount of urination
5. Shortness of breath	14. Hypochondriac pain or feeling of distension	23. Appetite	32. Distension of jugular vein
6. Gasp for breath	15. Edema	24. Sense of thirstiness	33. Cyanosis of face and lips
7. Posture	16. Tiredness	25. Loss of sleep/insomnia	34. Color of face/complexion
8. Coughing	17. Sweating at daytime	26. Dreaming	35. Color of lips
9. Phlegm in throat with sound	18. Sweating at night time	27. Somnolence	36. Location of edema

All enrolled patients are required to complete this questionnaire in a total of 6 specific study visits to the hospitals (i.e. Visit 1 at (Day 0), Visit 2(1 M), Visit 3(3 M), Visit 6(12 M), Visit 10(24 M) and Visit 14(36 M)/EOS). The visit schedule and list of parameters are shown in Table 3. For each scheduled visit, the physician/researcher will conduct a face-to-face interview with the patient to go through this survey questionnaire. The entire interview process will last for approximately 8 to 10 min. The data collected will be first recorded manually in the paper SDQHF and then input into the Epidata software [43] which is the centralized database for the collection of research data from the QUEST study. A pilot study with at least 600 patients will be conducted to evaluate the reliability, validity and feasibility of the modified TCM questionnaire for syndrome differentiation of HF.

**Table 3:** Planned visits and parameters

	Enrollment and allocation	Postallocation													Closeout	
Visit schedule	1	2	3	4	5	6	7	8	9	10	11	12	13	14	UNS	EOS
Day/month	Day0	1 M	3 M	6 M	9 M	12 M	15 M	18 M	21 M	24 M	27 M	30 M	33 M	36 M		
	− 14	± 3	± 7	± 7	± 7	± 7	± 7	± 7	± 7	± 7	± 7	± 7	± 7	± 7		= 2wks
Informed consent	x															
Inclusion/exclusion criteria	x															
Randomization	x															
General data and medical history	x															
Medical history of HF	x															
Medical history of CV diseases	x															
Physical examination	x	x^1^	x^1^	x^1^	x^1^	x	x^1^	x^1^	x^1^	x	x^1^	x^1^	x^1^	x	(x)	x
Heart failure medications	x	x	x	x	x	x	x	x	x	x	x	x	x	x	(x)	x
Medications of other CVD	x	x	x	x	x	x	x	x	x	x	x	x	x	x	(x)	x
Other medications	x	x	x	x	x	x	x	x	x	x	x	x	x	x	(x)	x
TCM Syndrome Diagnosis Questionnaire (SDQHF)	x	x	x			x				x				x		x
Tongue Image Acquisition	x	x	x			x				x				x		x
Echocardiogram	x^*^															
Pregnancy tests	x					x				x				x	(x)	x
Blood/urine routine test	x	x				x				x				x	(x)	x
Biochemical test	x	x				x				x				x	(x)	x
12-lead ECG	x^*^	x				x				x				x	(x)	x
Serum NT-proBNP at local laboratory	x	x		x												
Dispensing IP	x		x	x	x	x	x	x	x	x	x	x	x	x		
Returning IP for accountability			x	x	x	x	x	x	x	x	x	x	x	x		x
Endpoint event		x	x	x	x	x	x	x	x	x	x	x	x	x	(x)	x
Adverse event		x	x	x	x	x	x	x	x	x	x	x	x	x	(x)	x
Serious adverse event		x	x	x	x	x	x	x	x	x	x	x	x	x	(x)	x

X^1^ Simplified physical examination

x* Cardiac ultrasound and 12-lead ECG within the first 6 months of enrollment

UNS (unplanned visit): (x) The marked item is optional and performed according to the judgment of researchers

EOS (final visit): Make arrangement according to the study end time (if there is a visit within one month before the end of study, it is regarded as a final visit, but needs to be supplemented with the items required completely)

Pregnancy test is only applicable to women of childbearing age (if the urine pregnancy test is positive, it must be confirmed by serum pregnancy test)

### TCM syndrome differentiation

**Fig. 2:**
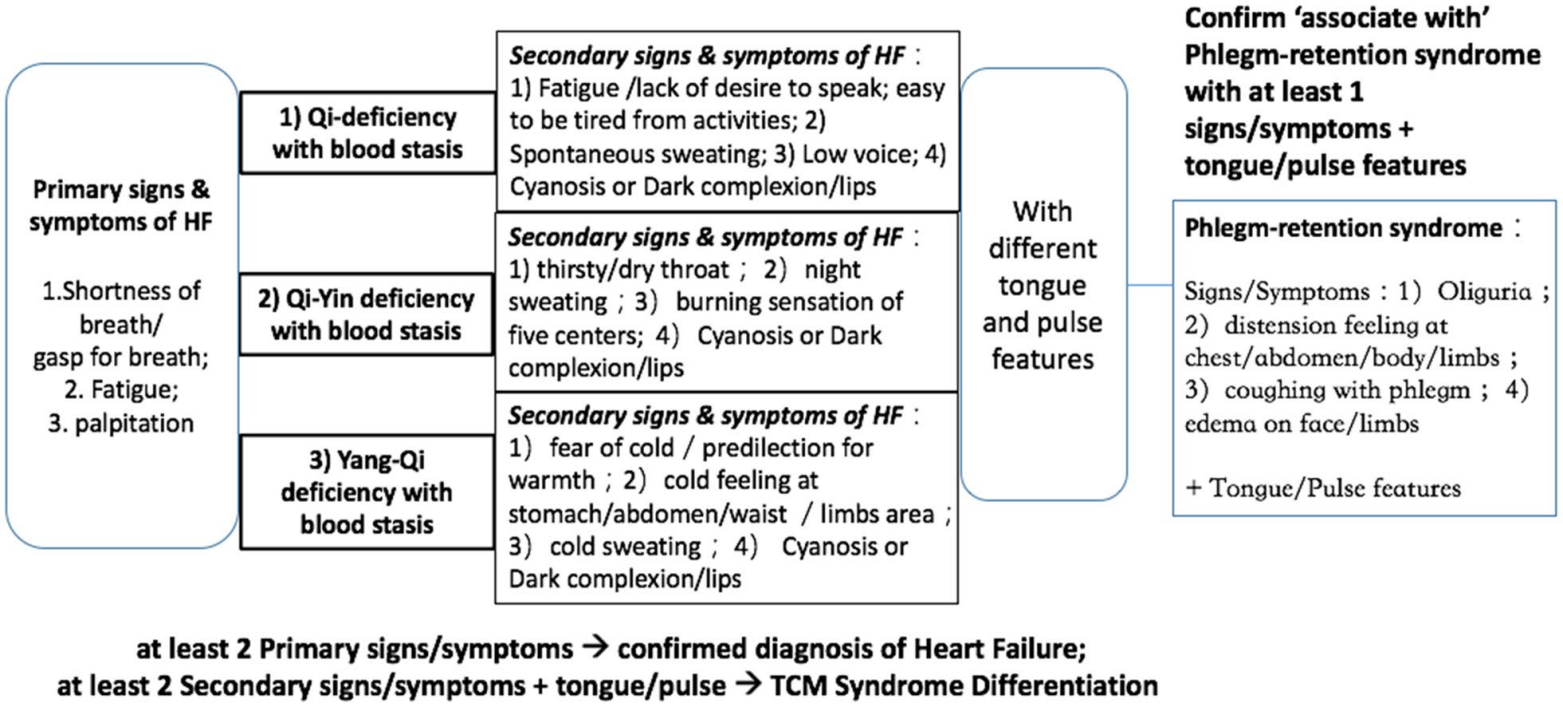
Diagnosis and TCM Syndrome Differentiation for Heart Failure according to the “2014 Consensus from TCM experts on diagnosis and treatment of chronic heart failure”

According to the “2014 Consensus from TCM experts on diagnosis and treatment of chronic heart failure” [17], there are three major types of TCM syndrome for heart failure: (1) qi-deficiency with blood stasis syndrome; (2) qi-yin deficiency with blood stasis syndrome; (3) yang-qi deficiency with blood stasis syndrome, and each can be associated with the phlegm-retention syndrome. These syndromes are comprised of a combination of 2–4 syndrome elements including (1) qi-deficiency; (2) yin-deficiency, (3) yang-deficiency; (4) blood stasis and (5) phlegm-retention. The list of clinical manifestations including signs and symptoms, pulse, and tongue features of the 3 TCM syndrome types are shown in Fig. 2. The 5 syndrome elements with 3 syndrome types indicated in this guideline are impeccably matched with the TCM Theory of Collateral Disease for heart failure.

A scoring model for syndrome differentiation is developed with reference to the criteria listed in Fig. 2 and the TCM theories. The output from the scoring model will be the type of TCM syndrome and the level of severity of heart failure patients at a specific stage of the disease. The output will be used for further analysis on the correlation of syndrome differentiation to the biomedical indicators, the severity of HF, and the therapeutic outcome of QLQX capsules.

### Correlation analysis of TCM syndrome patterns and the QLQX therapeutic outcome in CHF

Our primary objective is to explore the implication of TCM syndrome to the severity of heart failure and the therapeutic outcome of QLQX capsules. We hypothesize that the efficacy of QLQX Capsule on heart failure could be related to the clinical syndrome patterns in TCM diagnosis and likely be favorable to the patients with qi-deficiency with blood stasis and yang-qi deficiency with blood stasis.

The efficacy of QLQX capsule will be evaluated with reference to the “2012 Guiding Principles for Clinical Study on New Drug for Use in Traditional Chinese Medicine”. We will evaluate the correlation between TCM syndrome patterns and the severity of heart failure the re-hospitalization rates and mortality. The distribution of TCM syndrome will also be evaluated by NYHA classification, results from an electrocardiogram (ECG), cardiac ultrasound interpretation, and biochemical parameters from routine blood tesst such as NT-proBNP, urine test, etc. Other outcomes include: (1) the distribution of TCM syndrome patterns in heart failure patients; (2) the correlation between TCM syndrome patterns and the severity of heart failure and (3) the dynamic changes between biomedical parameters and the TCM syndrome patterns of heart failure, etc.

### Correlation of tongue features and the severity of HF and therapeutic outcome of QLQX

Tongue images of the patients will be acquired as part of the TCM Syndrome Diagnosis Questionnaire for Heart Failure (SDQHF) during the study visit. To ensure the quality and consistency of tongue photos from all clinical centers/hospitals, all researchers/physicians are properly trained according to the Standard Operation Procedures (SOP), and are provided with a standardized image acquisition device to capture the tongue image. The SOP includes the schedule of the study visit, the procedures for completing the SDQHF, and the criteria for capturing the tongue image such as the lighting requirement, camera mode (i.e. normal mode, no flashes, etc.), the posture of the tongue, and the tongue area to be included in the image, as well as the prerequisite requirements such as no intake of food or drinks that will stain the tongue coating on the day of the study visit. All tongue images should be sent to the central tongue image database via email with an indication of the patient number and the date of study visit.

We will set up a team of 4 TCM practitioners with 3 to 30 years of clinical experience to evaluate the tongue images. The identifiers and possible values for tongue analysis include the tongue color, tongue shape, tongue coating proper and its color, tongue coating distribution, appearance and location of ecchymosis, etc. Tongue images that fail to meet the SOP requirement will be excluded from the analysis. A 4-stage decision flow is designed for the review and analysis of tongue images: (1) direct confirmation if consistent interpretation and diagnosis from the first three TCM practitioners; (2) if consistent results are identical from 2 TCM practitioners but different diagnosis from the 3rd one, take the result from the majority; (3) if the interpretation and diagnostic results from all 3 TCM practitioners are different, will involve the 4th TCM practitioner with over 30 years of clinical experience to judge the syndrome type with the clinical information collected from the SDQHF; and finally, ‘94) if the image is determined as not in good quality for drawing the conclusion after reviewed by the separate TCM practitioner and professor, that tongue image will be excluded from the study.

Outcomes of the tongue analysis include (1) determination of the TCM syndrome differentiation of patients throughout the assessment period with the clinical information collected through the SDQHF; (2) tongue features in heart failure patients in accordance to the TCM syndrome, NYHA classification, and other biomedical data; (3) dynamic patterns of tongue condition in relation to the treatment efficacy and other biomedical indicators.

### Data management

The patient’s demographic data and all clinical data will be recorded and kept in the physical medical record folder during each study visit. The data will then be input into the Epidata software for centralization of all research data and all the tongue images will be kept in a separate centralized image database. The data management process will be complied with the regulatory requirements of Clinical Trial Quality Management Regulations and Clinical Trial Data Management Work Technical Guidelines to ensure the authenticity, integrality, accuracy, and traceability of data.

### Sample size estimations

This study is benchmarked with the QUEST study in which 3080 patients will be enrolled in over 130 centers (1540 patients per group) and followed up for at least 12 months. The sample size was estimated referring to the PARADIGM-HF study [44]. For QUEST study, the estimated incidence of cardiovascular death and hospitalization for heart failure is 25% in all patients receiving basic treatment + placebo group within 36 months of follow-up and 20% with those receiving basic treatment + QLQX Capsule group. The random distribution ratio is 1:1 between the study group and the control group. With the consumption of type I error in the interim analysis, α is adjusted to unilateral 0.02314. The sample size is the number of cases with composite endpoints events. Thus, 620 composite endpoint events are expected for the QUEST study to provide 80% power of test (β = 0.2), and the 20% risk can be reduced in the study group by the log-rank test.

For the current study, a pilot test with at least 600 patients will be conducted to evaluate the acceptability, reliability, and validity of the questionnaire. The number of patients for pilot testing is calculated based on the confidence level of 95%, population size of 3,080, and 5% margin of error for this 36-item questionnaire.

### Data analysis and statistic methodology

Analysis of the TCM Questionnaire will be performed with the SPSS statistical software package, version SPSS 24.0 (Chicago, IL, USA). Data will be presented by Mean ± S.D. Reliability Analysis and Principal Component Analysis (PCA) will be applied to examine the internal consistency and the factor structure of the questionnaire. Logistic regression analysis will be used to evaluate the capability of SDQHF to diagnose the severity of HF from a functional perspective. Pearson’s correlation coefficient will be used to determine the existence of correlations between TCM syndrome differentiation and various parameters such as treatment efficacy, NT-proBNP, NYHA classification, etc. An independent t-test will be used to compare quantitative variables for the two-group designed study. Fisher’s Exact test and the chi-squared test will be applied to compare categorical variables between groups. Multiple groups designed data will be analyzed by two-way ANOVA.

## Discussion

This is a multi-center, randomized, double-blind, parallel-group, placebo-controlled clinical trial to prospectively investigate the impacts of TCM syndrome patterns on the severity of heart failure and the therapeutic outcome of QLQX capsules. In the previous study, with 512 CHF patients and 12 weeks assessment period, QLQX capsules reduced the level of NT-proBNP and improved 6 min walk distances and quality of life [28]. Though the QLQX group had fewer deaths and re-admission incidence than the placebo group, the mortality rates were not significantly different [34]. Thus, we have moved forward to conduct the QUEST study, a large cohort clinical trial with 3080 HF patients from over 130 hospitals, and with a long follow-up period as well as hard endpoints for the long-term efficacy and safety of QLQX. It is expected to prove that QLQX could be an adjunct therapy with conventional treatment to reduce the mortality and re-hospitalization with worsening heart failure in the QUEST study.

However, this clinical trial does not include the study of TCM syndrome differentiations. According to the TCM theory, QLQX could tonify Qi and warming Yang to induce diuresis and dispel blood stasis. Thus, we raise the questions whether TCM syndrome patterns are associated with the severity of heart failure as well as the therapeutic outcome of QLQX capsules. As such, we leverage the QUEST study and design a connected yet independent clinical trial with completely different research aims. With the different aims and roadmaps from QUEST, this study will use the integrative medicine approaches to evaluate roles of TCM syndrome patterns in the progress of HF and their impacts on the therapeutic outcome. We expect that different TCM syndrome patterns and tongue characteristics would be associated with the severity and progression of heart failure, as well as the efficacy of QLQX on therapeutic outcomes. This study will shed light on the necessity of TCM syndrome differentiation for the HF treatment and the prediction of prognosis.

In TCM, the syndrome pattern is a vital component in both diagnosis and treatment of disease. Syndrome elements, syndromes, phenotypic features, as well as disease, form an integral process in the diagnostic path. However, due to the lack of standardization of syndromes, many diversified syndrome types were found in previous studies, leading to difficulties in drawing sound conclusive outcomes and thus, hindering research development [13–17]. It is crucial to have an end-to-end approach with a clear and definitive set of syndromes for heart failure study. As such, the present study will also focus on the definitive and quantitative syndrome diagnosis criteria for heart failure, the scoring model of the SDQHF, and the establishment of the tongue image database. For the TCM syndrome pattern recognition, we aim to develop a scoring system to catalog the syndrome differentiation from the signs and symptoms collected from the TCM syndrome differentiation questionnaire for HF which make references to the “2012 Guiding Principles for Clinical Study on New Drug for Use in Traditional Chinese Medicine”, “2014 Consensus from TCM experts on diagnosis and treatment of chronic heart failure”, and “2017 Guideline for TCM Diagnosis and Treatment of Heart Failure (Chronic Heart Failure)”, etc. We expect the development of SDQHF and the scoring model could establish a solid ground for syndrome differentiation of heart failure to facilitate the evaluation of the efficacy of QLQX Capsule. With this large-scale clinical trial and multi-dimensional evaluations, we believe we could provide strong evidence for the first time that TCM syndrome differentiations have potential implications to the disease progression and therapeutic outcome of heart failure. This may also enable the paradigm shift from reactive to predictive, preventive, and personalized care of heart failure patients through the application of the TCM syndrome differentiation.

In terms of operational issues, quality control is one of the major challenges for such large-scale clinical studies involving 130 clinical centers and more than 300 researchers/physicians in China and Hong Kong. We have established standardized operating procedure (SOP) and provided extensive training to all researchers/physicians prior to the patient recruitments. With the onset, supplementary training and continuous review, we could ensure proper execution of SOP to secure the quality of data collection. Tongue image acquisition is one of the critical issues for quality control. As such, we provided an imaging capturing device of the same model to each clinical center and trained all the researchers/physicians on how to properly acquire the tongue image, including the camera settings, the lighting requirement, and other pre-requisite requirements for patients prior to the photo-taking, etc. To ensure the image quality, we have a constant quality check on all tongue photos so that feedback for improvement could be provided to the researchers/physicians on a timely basis. Thus, the quality of tongue images could meet our requirement for the analysis of tongue features and patterns of heart failure. Last but not least, we also put efforts into patient communication to ensure all patients have a thorough understanding of the trial so that they can strictly follow through the requirement and attend all scheduled visits.

## Conclusion

We expect this clinical trial to be the high standard and high-quality study to assess the impacts of TCM syndrome patterns on disease progression and the therapeutic outcome for chronic heart failure patients with QLQX Capsule treatment. 

## Data Availability

Not applicable.

## Abbreviations

6MWD: 6-Min walking distance
CHF: Chronic heart failure
HF: Heart failure
NYHA: New York Heart Association
QLQX: Qiliqiangxin capsule
LVMi: Left ventricular mass index
LVEF: Left ventricular ejection fraction
TCM: Traditional Chinese Medicine
SDQHF: Syndrome Diagnosis Questionnaire for Heart Failure
NT-proBNP: NT-proB-type Natriuertic Peptide
SOP: Standard operating procedures
